# Paeoniflorin protects diabetic mice against myocardial ischemic injury via the transient receptor potential vanilloid 1/calcitonin gene-related peptide pathway

**DOI:** 10.1186/s13578-016-0085-7

**Published:** 2016-06-01

**Authors:** Fei Han, Dongchen Zhou, Xiang Yin, Zewei Sun, Jie Han, Lifang Ye, Wengting Zhao, Yuanyuan Zhang, Zhen Wang, Liangrong Zheng

**Affiliations:** Department of Cardiology, the First Affiliated Hospital, College of Medicine, Zhejiang University, No. 79 Qingchun Road, Hangzhou, 310003 China

**Keywords:** Paeoniflorin, Diabetes mellitus, Myocardial ischemia, Neuroprotection, TRPV1, CGRP

## Abstract

**Background:**

Diabetes mellitus has multiple complications including neuropathy and increases cardiovascular events. Paeoniflorin (PF), a monoterpene glycoside, plays an essential role in neuroprotection and ischemic heart disease. In this study, we aimed to investigate the hypothesis that PF protects mice with diabetes mellitus against myocardial ischemic injury, and determine its associated mechanisms.

**Results:**

Myocardial infarction (MI) was generated in the streptozotocin-mediated diabetic mice, which were pretreated with either vehicle or PF, respectively. Myocardial infarct size, myocardial enzyme, cardiac function, circulating calcitonin gene-related peptide (CGRP) concentration, histological analysis and the expression of associated molecules were determined and compared among different experimental groups. Compared to diabetic hearts pretreated with vehicle, hearts pretreated with PF exhibited less tissue damage and better CGRP concentration in serum when subjected to myocardial ischemia. Transient receptor potential vanilloid 1(TRPV1) gene knockout attenuated PF-mediated cardioprotection. Moreover, a specific Ca^2+^/calmodulin-dependent protein kinase (CaMK) inhibitor, KN-93, increased tissue damage and decreased CGRP activity in serum. Meanwhile, pretreated with PF increased the phosphorylation of cAMP response element binding protein (CREB).

**Conclusions:**

Taken together, these findings demonstrate that PF protects diabetic mice against MI at least partially via the TRPV1/CaMK/CREB/CGRP signaling pathway.

## Background

Diabetes mellitus (DM) has become a global problem that threatens human health [1, 2]. DM is characterized by increased plasma glucose levels and is often accompanied by several complications including microangiopathy, neuropathy and mobilopathy, which lead to high morbidity and mortality [3–5]. High blood glucose also impairs nerve fibers such as the sensory nerve fiber, which can give rise to painless myocardial infarction and reduce synthesis and release of neuropeptide; subsequently resulting in increased morbidity related to cardiovascular events and morbidity of sudden cardiac death (SCD) [6].

Transient receptor potential vanilloid 1 (TRPV1) is a non-selective cation channel that is mainly expressed in sensory neurons. Pharmacological studies have shown that TRPV1 exerts an important cardioprotective effect [7–9]. Previous studies have indicated that TRPV1 and its main neuropeptides calcitonin gene-related peptide (CGRP) and substance P significantly decreased in diabetic hearts, which was related to the poor recovery of cardiac function after myocardial ischemia [10, 11]. CGRP, the major neuropeptide released from nerve terminals, has both positive inotropic and potent vasodilatory effects which lead to augment post-ischemic cardiac performance [12]. Moreover preconditioning with CGRP protected against ischemia/reperfusion (I/R) injury in isolated hearts of rats [11].

The activation of TRPV1 by physical and/or chemical stimuli induces Ca^2+^ influx into neurons, leading to the activation of Ca^2+^-mediated signal transduction including the activation of Ca^2+^/calmodulin-dependent protein kinase (CaMK) [13–15]. CaMK is a multifunctional serine/threonine family with four isoforms, including Ca^2+^/calmodulin-dependent protein kinase II **(**CaMKII). A number of studies have reported that CaMK signaling is critical for a variety of neuronal functions. One of the downstream molecules of CaMKII is the cAMP response element binding protein (CREB), which has been identified as a critical transcription factor in spatial memory formation [16–18]. Activation of CREB transcription by calcium and cAMP signals increased CRE-mediated gene expressions such as structural proteins, signaling enzymes, or growth factors [19–21]. Previous studies have indicated that the activation of TRPV1 leads to an up-regulation of CGRP via CaMK-CREB cascade [22–25].

Paeoniflorin (PF) is a monoterpene glycoside, and exhibits various pharmacological activities including anti-inflammatory [26–29], antioxidant [30, 31] and immunoregulatory activities [32–34]. Furthermore, previous studies have suggested that PF is a potential neuroprotective agent. For instance, PF protected rats in a cerebral ischemia model [35–37]. PF attenuated the nerve injury and regulated the neurotransmitter release [29, 38, 39]. More recently, PF has been shown to attenuate acute myocardial infarction in a rat model [40, 41]. However, whether PF has any effects on ischemic heart in a DM model remains uninvestigated.

In this study, we investigated whether PF could protect DM mouse heart from MI-induced injury using an experimental MI model in DM mice. We found that PF protected diabetic hearts from MI-induced injury. We also provided evidence that the protective effects of PF on MI-induced cardiac injury was mainly via the TRPV1/CaMK/CREB/CGRP signaling pathway. Our findings provide novel insights into the molecular basis underlying PF-induced cardiac protection against ischemia in a DM model.

## Results and discussion

### Body weight and plasma glucose

Eight weeks after STZ injection, body weight and plasma glucose were measured to select the appropriate mice, and they showed no significant difference among the groups before surgery (data not shown).

### PF attenuates MI-induced cardiac injury

**Fig. 1:**
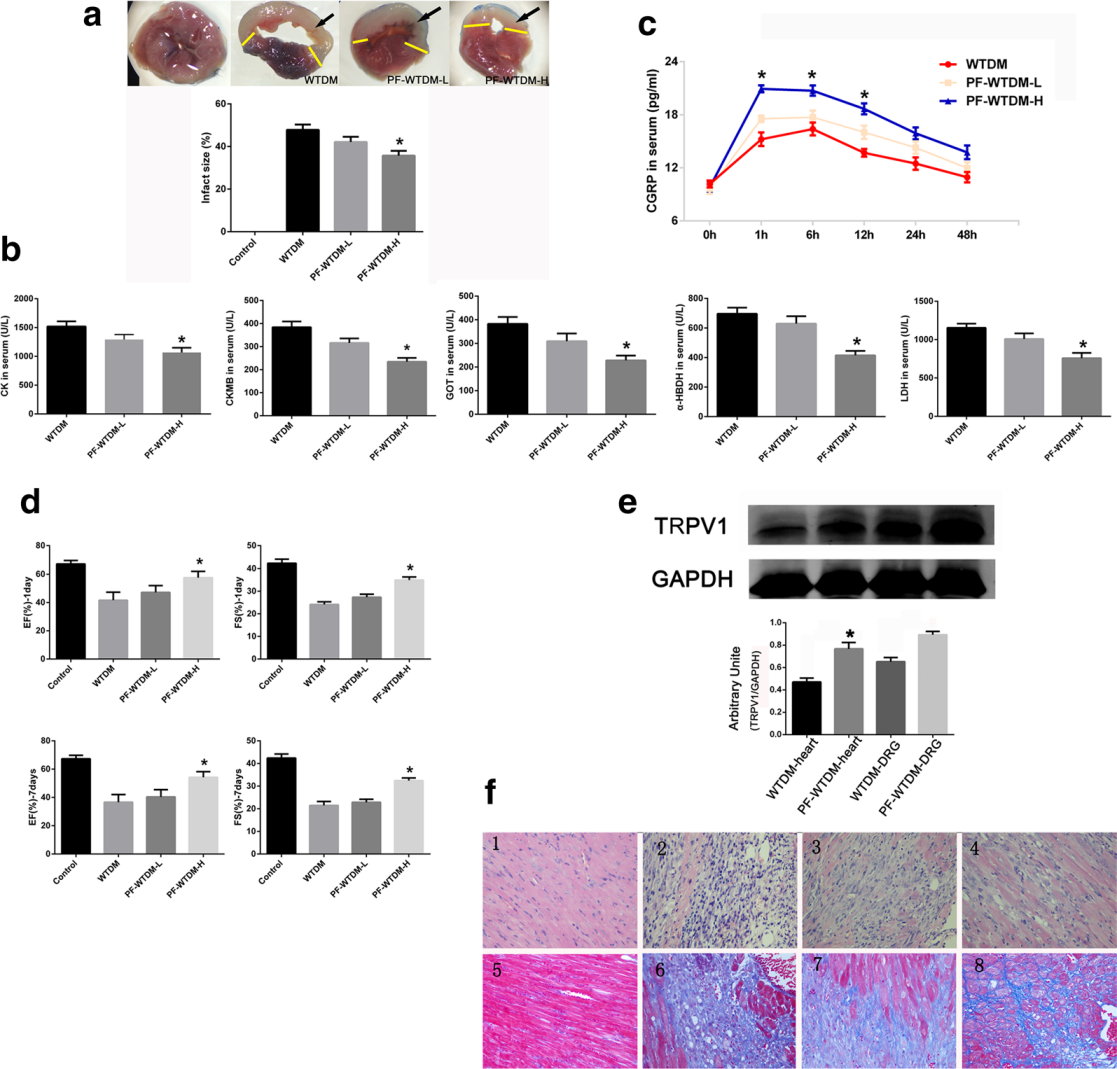
Paeoniflorin (PF) protects the heart against MI-induced injury in DM mice. **a** PF decreased myocardial infarct size. The *black arrow* indicates the infarction site. **b** PF decreased the release of myocardial enzymes in serum, including CK, CKMB, GOT, a-HBDH, and LDH. **c** the activity of CGRP in serum measured at different time points before and after MI. **d** Heart function was analyzed by measuring fractional shortening {FS = [LV end-diastolic diameter (LVEDD) − LVend-systolic diameter (LVESD)] × 100/LVEDD} and LV ejection fraction [LVEF = (LVEDD^2^ − LVESD^2^)/LVEDD^2^]. **d** Representative immunoblots of TRPV1 expression are shown. GAPDH was used as control. The *lower panel* shows the statistical analysis of the *upper panel*; *P < 0.05. **f** Representative images of hematoxylin and eosin (*1*–*4*) and Masson’s trichrome staining (*5*–*8*); (*1* and *5*) normal group (*2* and *6*) WTDM group (*3* and *7*) PF-WTDM-L group (*4* and *8*) PF-WTDM-H group. WTDM group: WTDM mice were pretreated with saline, PF-WTDM-L group: WTDM mice were pretreated with PF (70 mg/kg) before surgery, and PF-WTDM-H group: WTDM mice were pretreated with PF (140 mg/kg) before surgery (*n* = 6 per group; *P < 0.01, PF-WTDM-H vs. WTDM)

As shown in Fig. 1a, infarct size in mice treated with 70 or 140 mg/kg of PF for 14 days was smaller, compared with mice in the WTDM group. Although there was no statistical difference between the PF-WTDM-L and WTDM groups in terms of infarct size, a high dose of PF (140 mg/kg) significantly reduced infarct size compared with the WTDM group. During myocardial infarction, degradation of membrane integrity leads to the leakage of myocardial enzymes into the serum. Thus, the concentration of certain myocardial enzymes in serum reflects the severity of myocardial tissue injury. We measured some myocardial enzymes such as CK, CKMB, GOT, α-HBDH and LDH; and found that these enzymes were elevated in the serum of DM mice in vehicle, PF-WTDM-L and PF-WTDM-H groups. As shown in Fig. 1b, a PF dose of 140 mg/kg significantly decreased the levels of all these tested enzymes (Fig. 1b). Next, we measured the concentration of CGRP in the serum of mice in each group before and after MI surgery at different time points, as indicated in Fig. 1c. There was no difference in the activity of CGRP at baseline between each group. However, CGRP levels remarkably increased in each group subjected to ischemia; which peaked at 6 h after MI (*P* < 0.05). PF significantly enhanced the release of CGRP. Furthermore, PF significantly improved cardiac function (EF and FS) at day 1 and 7 after surgery, compared with the WTDM group (Fig. 1d). Next, we probed whether PF could change expression of TRPV1 by performing Western Blots on protein lysates purified from both the left ventricle and dorsal root ganglion (DRG) of mice in the WTDM and PF-WTDM-H groups, respectively. As shown in Fig. 1e, PF treatment significantly induced TRPV1 expressions in both the heart and DRG. Collagen production and deposition were evaluated by Massion’s trichrome staining of histological sections of the hearts. A high dose of PF strikingly reduced the interstitial fibrotic area in hearts compared with the WTDM group (Fig. 1f). These results indicate that pretreatment with PF significantly elevates the concentration of CGRP in plasma, which is a protective factor for diabetic hearts against MI-induced injury.

### TRPV1 gene knockout partially suppresses PF-mediated cardioprotection against MI

**Fig. 2:**
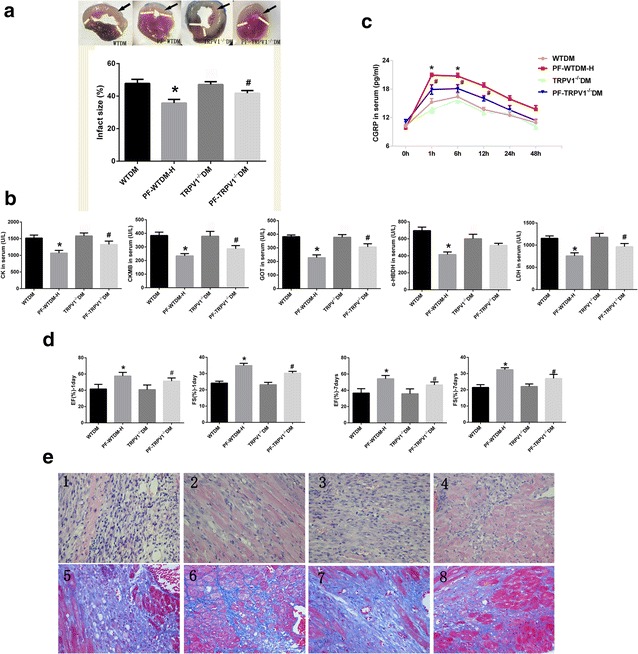
TRPV1 gene knockout partially abrogates paeoniflorin (PF)-mediated cardioprotection against MI. **a** TRPV1 knockout attenuated the decrease in myocardial infarct size by PF treatment, the *black arrow* indicates the infarction site. **b** TRPV1 knockout repressed the beneficial effects of PF on the release of myocardial enzymes in serum; **c** the activity of CGRP in serum measured at different time points before and after myocardial ischemia. **d** Heart function was assessed by fractional shortening {FS = [LV end-diastolic diameter (LVEDD) − LVend-systolic diameter (LVESD)] × 100/LVEDD} and ejection fraction [LVEF = (LVEDD^2^ − LVESD^2^)/LVEDD^2^]. **e** Representative images of hematoxylin and eosin (H&E, *1*–*4*) and Masson’s trichrome staining (*5*–*8*); (*1* and *5*) WTDM group (*2* and *6*) PF-WTDM group, (*3* and *7*) TRPV1^−/−^DM group, (*4* and *8*) PF-TRPV1^−/−^DM. PF-WTDM group: WTDM mice were pretreated with PF (140 mg/kg) before surgery, and TRPV1^−/−^DM group: TRPV1 gene knockout mice with diabetes mellitus; PF-TRPV1^−/−^DM, TRPV1 gene knockout DM mice were pretreated with PF (140 mg/kg) before surgery; *n* = 6 per group; *P < 0.01, PF-TRPV1^−/−^WTDM vs. PF-WTDM-H; ^#^ *P* < 0.05, PF-TRPV1^−/−^DM vs. TRPV1^−/−^DM. (Mean ± SEM)

Next, we used TRPV1 gene knockout mice to explore whether TRPV1 was involved in PF-mediated protection against MI. TRPV1 gene knockout had no significant effect on the severity of myocardial injury, compared with the WTDM group (Fig. 2a). PF significantly decreased infarct size and myocardial enzyme levels in mice in both WTDM and TRPV1^−/−^DM groups (*P* < 0.05, Fig. 2a, b). However, knockout of TRPV1 reduced the effect of PF on both infarct size and serum myocardial concentration (*P* < 0.05, Fig. 2a, b). In addition, TRPV1^−/−^ hearts (PF-TRPV1^−/−^DM) released less CGRP, compared with hearts in the PF-WTDM-H group (Fig. 2c). As shown in Fig. 2d, PF improved cardiac function in both TRPV1^−/−^DM and WTDM mice. However, the cardioprotective effects of PF were significantly better in WTDM hearts than in TRPV1^−/−^ hearts. PF reduced the interstitial fibrotic area in both WTDM and TRPV1^−/−^DM hearts, but the area in PF-WTDM-H was less than PF-TRPV1^−/−^DM group (Fig. 2e). These findings suggest that PF-mediated myocardial protection is achieved at least in part via TRPV1.

### Blockade of CaMKII attenuates the protective effects of PF on MI-induced heart injury in DM mice

**Fig. 3:**
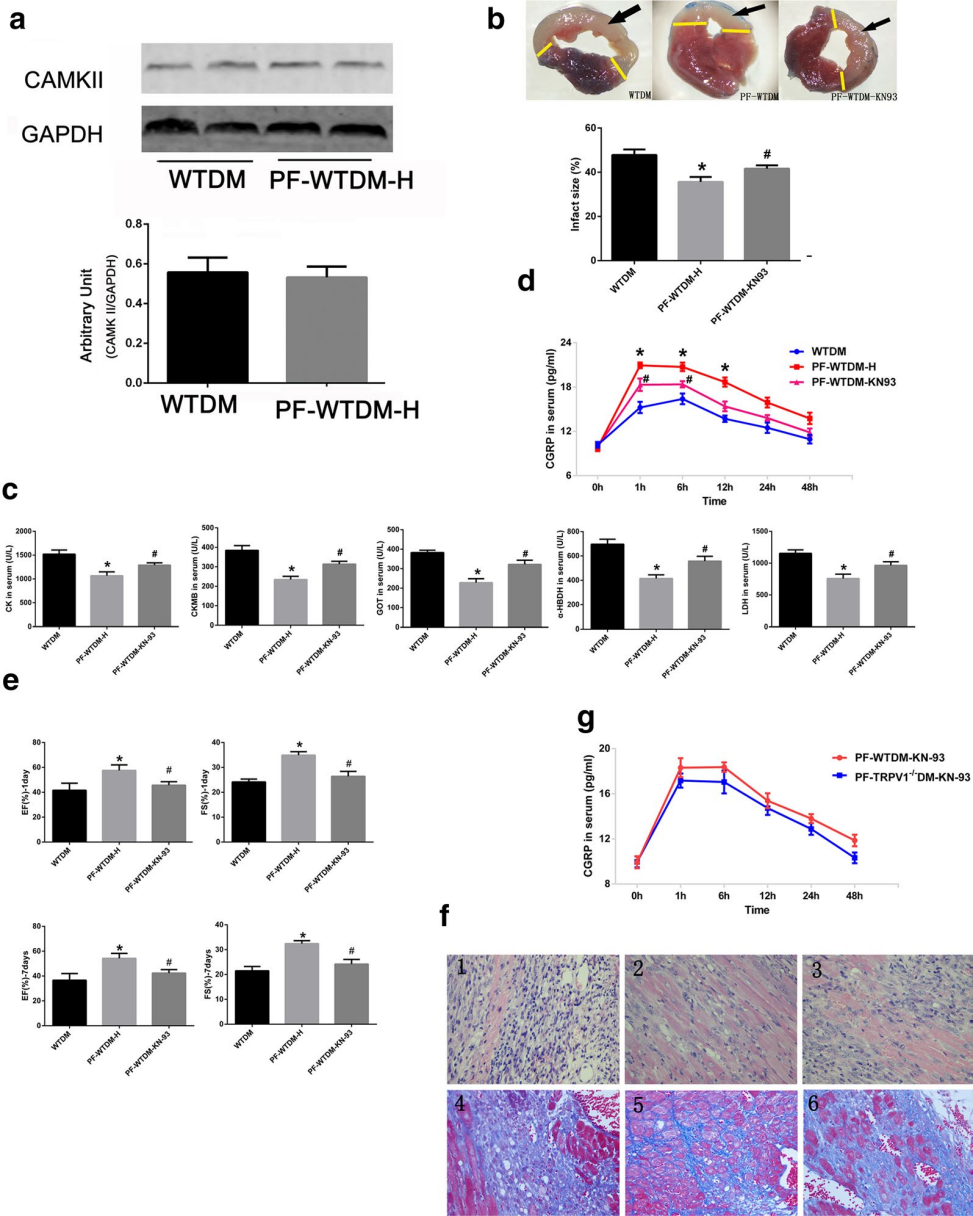
KN-93 attenuates the beneficial effects of paeoniflorin (PF) in DM mice. **a** PF treatment did not significantly alter CaMKII expression levels in hearts of mouse. The *lower panel* shows the statistical analysis results of the *upper panel*; GAPDH, *n* = 6/group. **b** KN-93 attenuated the PF-induced decrease in myocardial infarct size, the *black arrow* indicates the infarction site. **c** KN-93 inhibited the PF-repressed release of myocardial enzyme in serum. **d** KN-93 decreased the PF-induced increase in CGRP levels in serum at different time points after myocardial ischemia. **e** Heart function was assessed by fractional shortening and ejection fraction. **f** Representative images of hematoxylin and eosin (H&E, *1*–*3*) and Masson’s trichrome staining (*4*–*6*); *(1* and *4*) WTDM group, (*2* and *5*) PF-WTDM-H group, (*3* and *6*) PF-WTDM-KN-93. **g** CGRP levels in serum pretreated with PF in mice of the two groups as indicated. PF-WTDM-KN-93 group, PF-WTDM mice were pretreated with KN-93 at 10 min prior to coronary artery ligation; *n* = 6 per group. (*P < 0.05, PF-WTDM-H vs. WTDM; ^#^ *P* < 0.05, PF-WTDM-KN-93 vs. PF-WTDM-H)

We next measured CaMKII levels in the hearts of the above mentioned groups of mice, and did not observe any significant change between the two groups (Fig. 3a), its activity as a kinase might be involved in PF-mediated myocardial protection. KN-93, a selective CaMKII inhibitor, was used to test whether CaMKII activity is involved in PF-mediated myocardial protection. As shown in Fig. 3b and c, KN-93 significantly increased the concentration levels of circulating myocardial enzymes and infarct size in DM hearts, compared with vehicle-treated hearts. In addition, the release of CGRP significantly decreased when DM mice were pretreated with KN-93 (Fig. 3d). As shown in Fig. 3e, KN-93 attenuated the recovery of cardiac function, compared with the PF-WTDM-H group. Meanwhile, KN-93 also increased the interstitial fibrosis area compared with PF-WTDM-H group (Fig. 3f). These results indicate that CaMKII plays an important role in PF-mediated cardioprotection. Furthermore, TRPV1 gene knockout did not synergize with KN-93 to increase infarct size and release myocardial enzymes (data not shown) and the concentration of CGRP in serum were similar (Fig. 3g). Thus, we conclude that CaMKII is a downstream of TRPV1.

### PF increases the phosphorylation of CREB (pCREB)

**Fig. 4:**
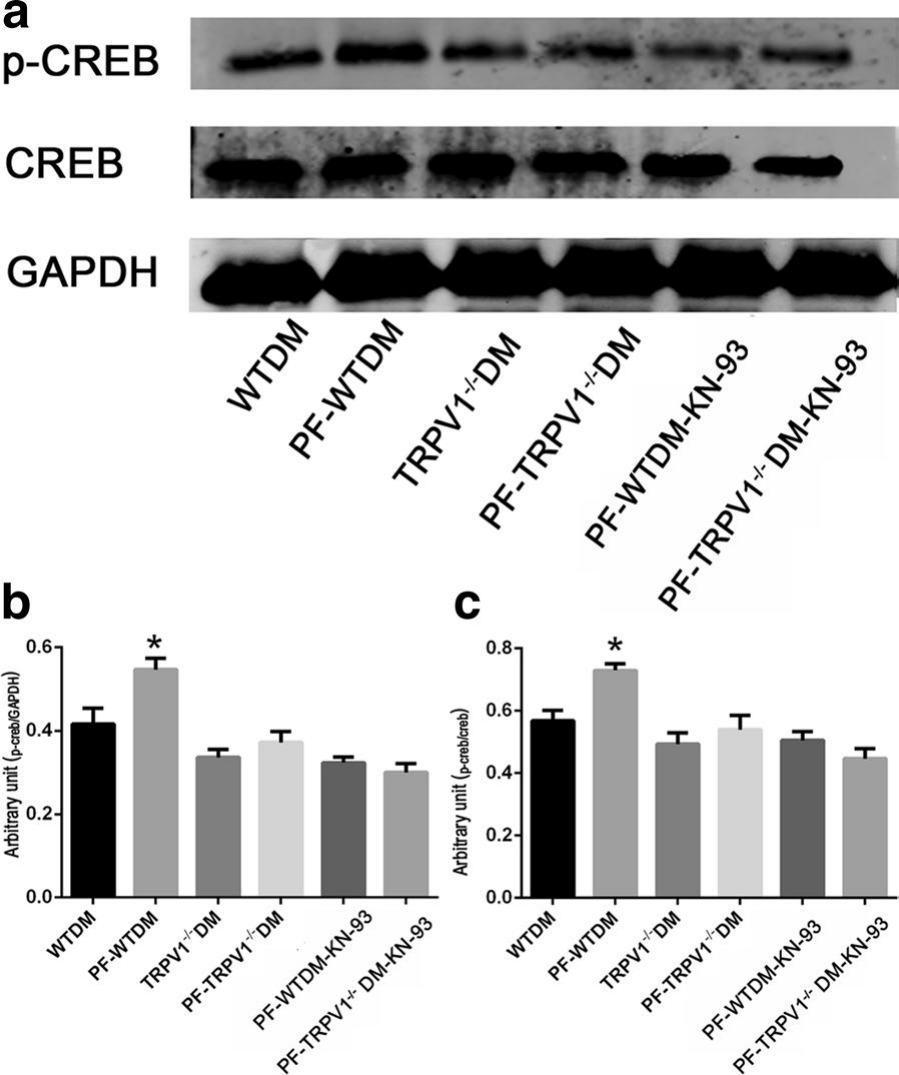
Paeoniflorin (PF) increases the phosphorylation of CREB (pCREB). The level of pCREB was significantly higher in the PF-WTDM group than in the other groups. **a** A representative image of the levels of pCREB, CREB and GAPDH. **b** Quantification by the ratio of pCREB to GAPDH (*n* = 6/group). **c** Quantification by the ratio of pCREB to CREB. *P < 0.05, PF-WTDM-H vs. WTDM, TRPV1^−/−^DM, PF-TRPV1^−/−^DM, PF-WTDM-KN-93 and PF-TRPV1^−/−^DM-KN-93

Previous studies have shown that pCREB regulated CGRP levels [42]. Thus, we determined whether PF could increase the release of CGRP via increased pCREB. pCREB levels in PF-DM hearts were significantly higher than in control mice. Both TRPV1 gene knockout and KN-93 suppressed the levels of pCREB. Taken together (Fig. 4), these findings indicate that PF potentiates pCREB; which can be blocked by either TRPV1 gene knockout or the CaMKII inhibitor.

## Discussion

The main findings of the present study are as follows: First, PF indeed protected the DM heart against MI-induced injury, as evidenced by reduced myocardial infarct size, decreased myocardial enzyme release, improved cardiac function recovery, increased the expression of TRPV1 and release of CGRP in serum in PF-pretreated diabetic mice; second, TRPV1 gene knockout partially suppressed PF-mediated cardioprotection; third, KN-93, a specific inhibitor for CaMKII, attenuated the protective role of PF in wild type DM mice, and did not change the CGRP concentration in TRPV1 gene knockout mice, which is consistent with the notion that CaMKII is a downstream of TRPV1; fourth, PF-mediated cardioprotection was related to the phosphorylation of CREB.

Coronary artery disease remains the leading cause of death in patients with DM. Diabetic mice displayed exacerbated injury following myocardial ischemia–reperfusion and are resistant to most therapeutic interventions [43, 44]. With the increasing number of diabetic patients and its complex complications, it is important to explore the application values of PF on diabetic mice with myocardial ischemia injury.

Myocardial ischemia is the leading cause of death worldwide, and a wealth of evidence points to the multiple mechanisms underlying the MI-induced injury including inflammatory and oxidative stress. Our previous researches have provided the evidence for a role of the neuropeptide in the progression of MI [7, 8]. Diabetic neuropathy, including central nervous and peripheral nerve, is one of the most frequent complications of DM and results in a poor prognosis and increased mortality, and the impaired afferent fibers that run in the cardiac sympathetic nerves play the essential role in silent myocardial ischemia in DM patients. The activation of TRPV1, which is expressed in sensory nerve fibers, could mediate the transmission of pain and activate the synthesis and release of cardiacprotective neuropeptides [12, 45]. Thus, TRPV1 was speculated to play a multiple part in DM mice subjected to myocardial ischemia. Our previous studies revealed that neuropathy was obvious after 8 weeks in the mice with STZ-induced DM, in which both TRPV1 and CGRP were impaired in DM heart and subsequently increased the MI-induced injury [10]. In present study, we showed that PF significantly attenuated the MI-induced injury. Meanwhile, we also found that PF reversed the impaired TRPV1 in DM mice, which is consistent with the neuroprotective function of PF. This study is the first to investigate the protective effects of PF on myocardial ischemia from the view of improving diabetes-induced nerve injury. Thus, our study provides evidence that PF may potentially have beneficial effects on DM patients with neuropathy or coronary heart disease.

TRPV1 is a Ca^2+^-permeable channel protein, and the activation of the TRPV1 channel increases the concentration of intracellular Ca^2+^ and, in turn, activates CaMKII. The activation of CaMKII subsequently promotes the phosphorylation of CREB, which has an essential role in CaMK-mediated the up-regulation of CGRP levels [27]. In our study, we explored the role of TRPV1 in the PF’s cardioprotection in the TRPV1 gene knockout mice. We found that the PF pretreated TRPV1^−/−^DM mice showed worse heart injury than in WTDM mice, suggesting that TRPV1 gene knockout suppress PF-mediated cardioprotecion. In addition, the blockade of CaMKII by a specific inhibitor, KN-93, attenuated the effects of PF in WTDM mice, and this inhibition in TRPV1^−/−^DM mice did not significantly change the concentration of CGRP in serum, further demonstrating that CaMKII was a downstream of TRPV1 signaling. Another important finding is that PF promoted the phosphorylation of CREB, which was suppressed by TRPV1 gene knockout and CaMKII inhibitor. These findings support the views of the TRPV1-CaMKII-CREB signaling cascade [46]. Our study has shed light into the mechanisms underpinning PF-induced cardiac protection in DM mice subjected to myocardial ischemia, and provides rationales to support further studies in our traditional Chinese medicine.

Several limitations in our study need to be pointed out. First, we only explored PF-mediated TRPV1 changes in the heart and DRG, and whether PF can offer neuroprotection needs further studies. Second, our study revealed that PF protected TRPV1^−/−^DM hearts, indicative of the involvement of other pathway(s) in PF-mediated cardioprotection in DM heart. Third, the exact mechanisms by which PF improved cardiac function remains unclear, and this may be achieved via either the direct interaction of PF with TRPV1, or the anti-inflammatory and anti-oxidative mechanisms of PF, or both. Fourth, although we provide evidence showing that PF may represent a potential new therapeutics for DM patients with coronary heart disease or neuropathy, clinical trial needs to be carried out for its clinical application.

## Conclusions

In conclusion, our study demonstrates that PF provides cardioprotection against MI-induced injury at least in part via increasing the activity of CGRP in serum in DM mice. We have also provided evidence that the TRPV1/CaMKII/pCREB signaling pathway is involved in PF-mediated myocardial protection.

## Methods

### Materials and methods

#### Materials

Antibodies against TRPV1 and GAPDH were obtained from Cell Signaling Technology (Boston. USA); PF was purchased from Zeheng corporation (Hangzhou, China); anti-CaMKII was obtained from Abcam (UK); anti-CREB and anti-pCREB were obtained from CST (USA); Evans blue was purchased from Sigma–Aldrich (Saint Louis, MO, USA); and 2,3,5-triphenyltetrazolium chloride (TTC) was purchased from Biosharp (Anhui, China). KN-93 was purchased from Selleck (USA).

#### Animals

TRPV1 gene knockout mouse was obtained from the Jackson Laboratory (Bar Harbor, ME, USA), and TRPV1^−/−^ genotype was confirmed by PCR based on the protocol provided by the Jackson Laboratory. Matching control WT strain C57BL/6J mice (Shanghai Laboratory Animal Center of the Chinese Academy of Sciences), weighing 20–22 g, were used in this study. Mice were housed under a standard SPF environment with a 12-h dark-light cycle, and free access to water and food were provided, as previously reported [47]. All animal experiments were conducted in accordance with the Guidelines for the Care and Use of Laboratory Animals, and were approved by the Animal Ethics Review Committee of Zhejiang University.

#### Experimental protocols

**Fig. 5:**
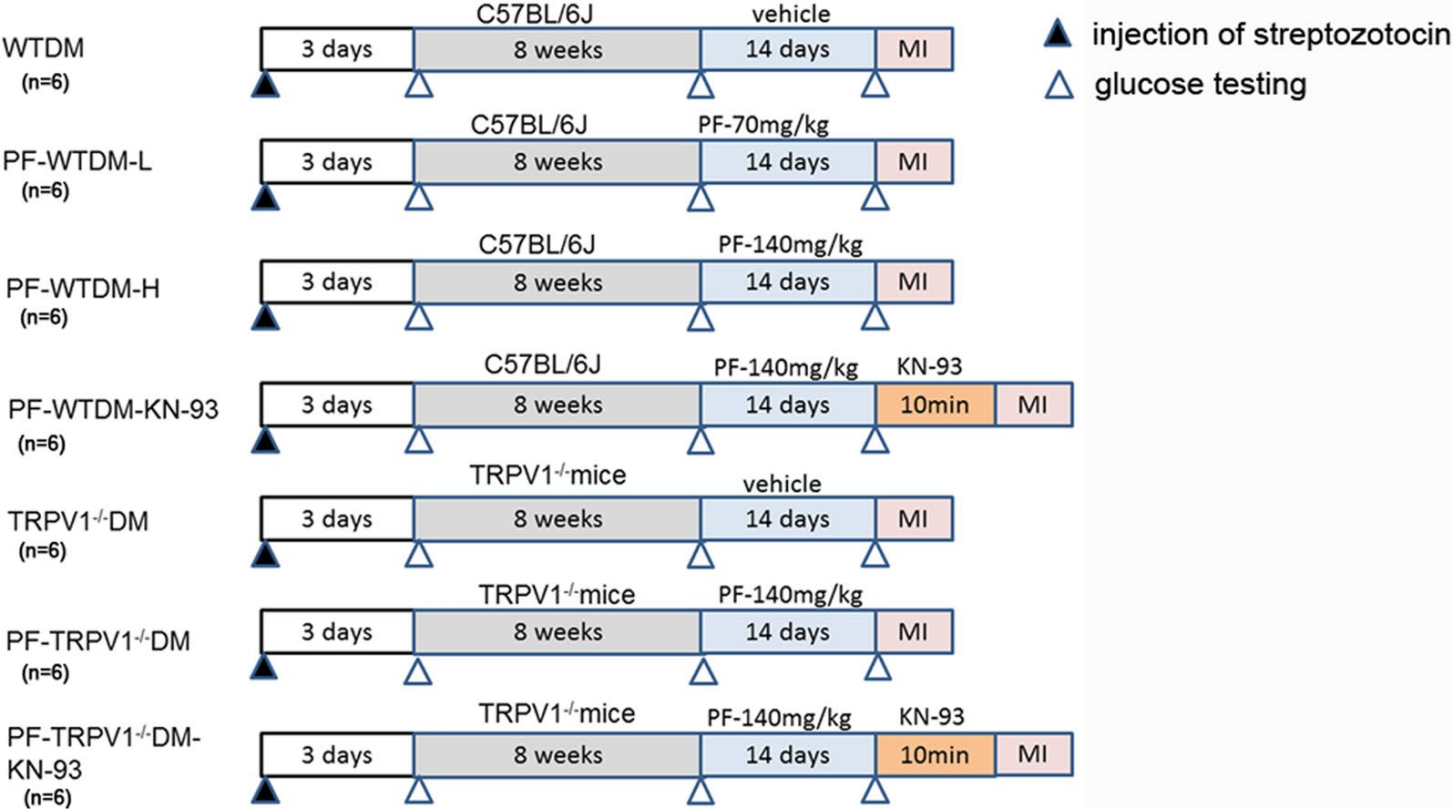
Detailed experimental protocols used in the present study. The animals were divided into seven groups: (1) WTDM group: diabetes mellitus (DM) in wild type mice; (2) PF-WTDM-L group: WTDM treated with 70 mg/kg of paeoniflorin; (3) PF-WTDM-H WTDM treated with 140 mg/kg of PF; (4) PF-WTDM-KN-93 group: KN-93 was used to treat DM mice; (5) TRPV1^−/−^ DM: TRPV1^−/−^ mice was used for the DM model; (6) PF-TRPV1^−/−^ DM: TRPV1^−/−^ DM treated with 140 mg/kg of PF; (7) PF-TRPV1^−/−^ DM-KN-93 group, KN-93 was used to treat TRPV1^−/−^ DM mice (*n* = 6/group)

Male C57BL/6J and age-matched TRPV1 gene knockout mice were subjected to intraperitoneal injection with 150 mg/kg of streptozotocin (STZ) in a fasting state. A OneTouch^®^ SureStep™ Blood Glucose Monitoring System (LifeScan, USA) was used to measure plasma glucose levels at 3 days and 8 weeks post-STZ injection. A blood glucose concentration >16.7 mmol/l were up to the criteria at 3 days and 8 weeks for experimental DM mice [48]. Detailed experimental protocols are shown in Fig. 5. Basically, mice were randomly divided into seven groups: WTDM, PF-WTDM-L, PF-WTDM-H, TRPV1^−/−^DM, PF-TRPV1^−/−^DM,PF-WTDM-KN-93, and PF-TRPV1^−/−^DM-KN-93.

After DM mice were established based on the plasma and urine glucose levels mentioned above, they were given 70 or 140 mg/kg/day of PF for 14 days by the intragastric route; except the control group, which were treated with saline as previously described [49]. After drug treatment, an acute myocardial infarction animal model was induced by the permanent ligation of the left coronary artery. First, mice were anesthetized with urethane (1.0 g/kg, intraperitoneally). After left thoracotomy, the heart was exteriorized, and the left coronary artery was ligated proximately 2 mm from its origin between the pulmonary artery and left atrium with a 8–0 Prolene suture. Then, mice were housed in conventional cages with free access to water and rodent chow.

After more than 14 days of PF treatment in mice, repeated boluses of KN-93(300 μg/kg), a selective CaMKII inhibitor (Selleck, USA), was administered directly into the left ventricle, 10 min before the anterior descending branch was blocked.

#### Measurement of myocardial infarct size

Twenty-four hours after coronary artery ligation, the chest was opened, and the heart was perfused with (1 %) Evans blue solution (2 ml), into the aorta and coronary arteries. The heart was then isolated when its beating was slow. After removing the right ventricle and atria, the left ventricle was sliced into 1.5-mm-thick cross-sections below the ligature. These segments were weighted, and incubated with 1 % TTC for 20 min at 37 °C in the dark. The non-ischemic area (Evans blue-stained) and ischemic area (TTC-unstained) were analyzed by the ImageJ (National Institutes of Health) [50]. Photographs of the infarct areas in the total left ventricular area were quantified as infarct area/left ventricle (INF/LV) using ImageJ [8].

#### Determination for myocardial enzyme in serum

Eyeballs were extracted and blood was collected into a test tube. The collected blood was centrifuged at 3000 r/min for 15 min at 4 °C, and the serum obtained was stored at −20 °C. Serum concentrations of myocardial enzymes including glutamic-oxaloacetic transaminase (GOT), creatine kinase (CK), creatine kinase-MB (CK-MB), lactate dehydrogenase (LDH), and α-hydroxybutyric dehydrogenase (α-HBDH) were measured using commercial kits (Nanjing Jiancheng Biotechnology Institute, Nanjin, China), according to manufacturer’s instructions.

#### Western blot

Cardiac tissues were lysed in RIPA lysis buffer, proteins were extracted, and protein concentrations were measured using the bicinchoninic acid method. Western blot was preformed as previously described [11]. The blot was probed with the primary antibody of interest as indicated in the Figures, where applicable; and coloration was visualized by the enhanced chemiluminescence kit (Thermo Scientific, Rockford, IL, USA). Specific protein bands were quantified and normalized to GAPDH.

#### Radioimmunoassay

Plasma was obtained at 0, 1, 6, 12, 24 and 48 h after surgery was performed on mice. The plasma was prepared as described before. CGRP concentration in plasma was measured with a commercially available rabbit anti-rat CGRP radioimmunoassay kit (Phoenix Pharmaceuticals) [51].

#### Echocardiography

Echocardiography was performed at day 1 and 7 after surgery, as previously detailed [52–54]. Mice were anesthetized and placed on a warm pad to maintain body temperature. Hair was removed from the upper abdominal and thoracic areas with depilatory cream [55, 56]. Two-dimensional echocardiography was performed using a high-resolution imaging system with a imaging transducer (GE Vingmed Ultrasound). Images were analyzed offline by a researcher blinded to the murine genotypes. Ejection fraction (EF) and fractional shortening (FS) were calculated from the M-mode parasternal short axis view [57, 58].

#### Histological evaluation

Histopathological changes and collagen distribution were estimated by haematoxylin and eosin (H&E) and Massion’s trichrome staining. The ventricles were fixed in 4 % paraformaldehyde, then embedded with paraffin and cut cross-sectionally into 5-µm thick sections. Tissue sections were deparaffinized and stained with H&E or Massion’s trichrome reagent. The particular procedure was described before [59]. The analysis of the picture was using ImageJ.

### Statistical analysis

Data are expressed as mean ± SEM. Statistical significance was determined by student’s *t* test. A *P* < 0.05 was considered statistically significant.

## Abbreviations

DM: diabetes mellitus
PF: paeoniflorin
MI: myocardial infarction
TRPV1: transient receptor potential vanilloid 1
CGRP: calcitonin gene-related peptide
CaMKII: Ca^2+^/calmodulin-dependent protein kinase II
CREB: cAMP response element binding protein
DRG: dorsal root ganglion
